# Magnitude of Child Food Insecurity, Its Association with Child Immunization and Huosehold wealth Status, and Coping Strategies In Dabat Demographic and Surveillance System North West Ethiopia

**DOI:** 10.1155/2020/3746354

**Published:** 2020-09-03

**Authors:** Nigusie Birhan Tebeje, Gashaw Andargie Biks, Solomon Mekonnen Abebe, Melike Endris Yesuf

**Affiliations:** ^1^School of Nursing, College of Medicine and Health Sciences, University of Gondar, Gondar, Ethiopia; ^2^Department of Health Service Management and Heath Economics, Institute of Public Health, College of Medicine and Health Sciences, University of Gondar, Gondar, Ethiopia; ^3^Department of Human Nutrition, Institute of Public Health, College of Medicine and Health Sciences, University of Gondar, Gondar, Ethiopia

## Abstract

**Background:**

The magnitude of food insecurity in Ethiopia ranges from 38.7% to 82.3% among the general population. Children under the age of five years were more prone to food insecurity and its serious consequences like anemia, low bone density, frequent episodes of common cold, stomachache, poor educational performance, and dental carries in developing countries like Ethiopia. However, there is no any research finding that documented the magnitude of child food insecurity, coping strategies, and associated factors in the study area. Therefore, the aim of this study was estimating the magnitude of child food insecurity, major coping strategies, and factors associated with child food insecurity in the study area.

**Methods:**

A community-based cross-sectional survey has been conducted in Dabat demographic and health surveillance site among 7152 mothers/caretakers of children under the age of five years. Data were collected by experienced data collectors working for the demographic and health surveillance site, and the collected data were entered into EpiData template and then transported to Stata 14 software for data cleaning and analysis. The ordinal logistic regression model was fitted to identify predictors for child food insecurity.

**Results:**

About 21.42% of children under the age of five years were food insecure in Dabat district of whom 57.8%, 38.6%, and 3.6% had experienced mild, moderate, and severe levels of child food insecurity, respectively. All most all 1391 (92%) of the mothers/caretakers of food insecure children had practiced food insecurity coping strategies. More than half (57%) of mothers/caretakers reduces the size of child meal as insecurity coping strategy. Child food insecurity was associated with household wealth status, parent's education status, and maternal and child health service utilization and child feeding practices.

**Conclusion:**

A large segment of under-five children had experienced food insecurity in Dabat district, and the major coping strategy for child food insecurity was reducing meal size. Therefore, working on household wealth improvement and expansion of basic health services would improve child food security.

## 1. Background

Food security is a concept that existed when all people at all times have physical and economic access to safe and sufficient food [1, 2]. Assuring food security for mankind is among the priority agendas of leaders around the world. United Nations sustainable development goal (SGD) has two targets dedicated for assurance of food security [2]. Despite these efforts globally, the prevalence of food insecurity was 9.3% in 2016 [3]. In Africa, it was a serious public health problem that 31% of its population were food insecured [4].

Risk factors for the increased magnitude of food insecurity like drought, conflict, pests, livestock diseases, corruption, political instability, AIDS, and rapid population growth were widely distributed in Africa. Children of Africa were even at greater risks of food insecurity and its risk factors [5]. And situations related to and with food insecurity in sub-Saharan Africa were more alarming.

In Ethiopia, the magnitude of food insecurity was estimated to range from 38.7% to 82.3% with a trend shifting from urban to rural households and was fueled by adverse climatic change [6–11]. The most recent climatic change “El Niño” has dropped 50-90% of crop production and makes 10.2 million Ethiopian people food insecure. In addition to this, 2.2 million farmers and herders need agricultural support during post El Niño in Ethiopia [12]. The effect of this El Niño could sustain in the future and children were at greater risks of food insecurity in different parts of Ethiopia. Similarly, poverty, one of the manifestation for food insecurity, was widely distributed among Ethiopian rural community in general and children under the age of five years in particular [13].

Serious health problems like developmental delays, iron deficiency anemia, less physical activity, low bone density, poor health-related quality of life, mental health problems, more frequent episodes of common cold and stomachaches, poor educational performance, and dental caries were associated with child food insecurity [14]. However, there is no study that documented the magnitude, coping strategies, and associated factors of child food insecurity in Ethiopia.

On the other hand, when we look at child malnutrition and feeding practice factors closely related with child food insecurity, 38%, 10%, and 24% of children under the age of five years were stunted, wasted, and underweighted, respectively, in Ethiopia. And only 7% and 14% of children aged between 6-23 months had received minimum acceptable dietary standards and adequately diversified diet [15].

Furthermore, child food insecurity leads to less socioeconomic development, increased health care cost, decreased income, and the sustained prevalence of child malnutrition in developing countries like Ethiopia but not investigated well [16]. But we have evidence even on the magnitude, coping strategies, and associated factors for child food insecurity [17–19]. Therefore, the main aim of this survey was to determine the prevalence child food insecurity, coping strategies, and associated factors in the study area.

## 2. Methods and Materials

### 2.1. Study Area

The study was conducted at Dabat Demographic and Health Surveillance System (HDSS) site. The HDSS covers 13 kebeles divided into 83 clusters. The altitude of the district ranges from 1000 meters to 2500 meters above sea level, and the weather condition is divided into highland, midland, and lowland climatic conditions. Dabat town, the capital of the district, is located 821 km from Addis Ababa and 75 km from Gondar town to the north. Dabat district has six health centers, three health stations, and thirty-one health posts that provide health services to the community. The total population of the district was estimated to be 158,250 of whom about 70,611 were the population of the HDSS with the sex ratio of nearly 1 : 1. In the HDSS, there are 7,918 children under the age of five years [20].

### 2.2. Study Design and Population

The community-based cross-sectional study design was carried out among rural and urban households with under-five children from April to December 2016. Mothers/caretakers of under-five children available during the study period were participants for this study. In the absence of mothers/caretakers of under-five children during visit day, other senior household members beyond 18 years were interviewed after consenting.

### 2.3. Data Collection Tool and Data Collection Procedure

A pretested interviewer-administered structured questionnaire was used to collect data on sociodemographic, and maternal and child health service utilization. To assess the level of child food security, we use FANTA III food insecurity access measurement scale version 3. A five-day training was provided for data collectors and supervisors. A pretest was conducted in the rural and urban kebeles of Dabat district which are not included in the HDSS, and necessary modification of the tool was made according to the inputs of the pretest. Data were collected by 15 experienced data collectors and supervised by supervisors working for Dabat HDSS.

### 2.4. Data Processing and Analysis

To avoid data entry errors related to the translation of the responses, collected data were entered into EpiData template prepared in Amharic language that have similar content with the data collection tool. Five experienced data entry clerks were recruited for the data entry, and the overall data entry process was supervised by a data manager working at the HDSS site. Entered data were transported to STATA version 14.1 for data cleaning and analysis. The correlation of a dependent variable with each independent variable was assessed by Pearson's chi-square test before fitting univariate and multivariate ordinal logistic regression models. All variables with significant Pearson's chi-square test were considered for univariate ordinal logistic regression model, and variables significant at the univariate ordinal logistic regression model were fitted into the multivariate ordinal logistic regression model to identify predictors for child food insecurity. Proportional cumulative odds ratio assumption was checked by significant Pearson's chi-square before the attempt to interpret the final model.

### 2.5. Study Variables

#### 2.5.1. Dependent Variable

The level of child food insecurity is the dependent variable.

#### 2.5.2. Independent Variables

The following are the independent variables:

- Sociodemographic characteristics: wealth status of the household, parents' educational status, marital status and residence of head of the household, number of children in the household, age of children in the household, sex of children in the household, family size, religion, ethnicity, and occupation of parents, and availability of garden source of food item for the household and child food preparation and feeding practices
- Maternal and child health utilization: TT vaccine, iron supplementation, ANC visit, place delivery for the last pregnancy, PNC service for the last birth, Vit A supplementation for children in household, child deworming, BCG, polio, DPT, Penta valiant, Rota, PCV, measles vaccine, illness, and treatment for illness

#### 2.5.3. Operational Definition

- Child food security: if the interviewed mothers/caretakers answered rarely only Q1a and not the rest of the questions
- Mild food insecurity: if the interviewed mothers/caretakers answered sometimes or often Q1a or rarely, sometimes or often Q2a, or rarely Q3a or Q4a
- Moderate food insecurity: if the interviewed mothers/caretakers answered sometimes or often Q3a or Q4a or rarely or sometimes Q5a or Q6a
- Severe food insecurity: if the interviewed mothers/caretakers answered often Q5a or Q6a or rarely or sometimes or often questions Q7a-Q8a

## 3. Result

### 3.1. Sociodemographic Characteristics of the Household and Under-Five Children in Dabat HDSS Site

A total of 7,152 mother/caretakers of under-five children have participated in this study, of whom 7061 (98.72%) have completely responded for the interview. More than half (53.49%) of heads of the household were unable to read and write, and 76.74% of them were married. Almost all (98.74%) of mother/caretakers of under-five children were Amhara in ethnicity and 96.11% orthodox Christians by religion. Half of under-five children (50.52%) were female, and almost a quarter (23.64%) of them were in the age category of 37-48 months. About 34.18% of children were at the fifth or above birth order and 37.77% of them have three-year birth interval (Table 1).

### 3.2. Health Service Utilization and Child Feeding Practice among Participants in Dabat HDSS Site

About 65.86% of mothers have history of ANC visit during their last pregnancy. The majority (69.91%) of mothers gives birth in home, and none of them had received postnatal care during the last delivery. Among mothers who have history of institutional delivery, 87.63% of the deliveries were occured in health centers. Almost all (95.67%) of children have received at least one dose of vaccination. As to child feeding practice, 58.58% of mother have history of initiating breast feeding within one hour of delivery, 59.35% of them have reported history of six months of exclusive breast feeding, and 57.84% of the mothers have introduced supplementary feeding for children at six months of age. On the other hand, 29.64% and 16.84% of the mothers had practiced discarding of colostrum and prelacteal feeding, respectively (Table 2).

### 3.3. Major Child Health Problems and Treatment Seeking within the Last Two Weeks of the Survey in Dabat HDSS

About 2193 (30.75%) of the mothers/caretakers of under-five children have reported child health problems, in the last two weeks of the survey, of whom half of 1128 (51.44%) them have experienced fever. Only 517 (23.57%) have treated for illnesses in health institutions, and more than half 301 (58.22%) of them were treated in health centers (Table 3).

### 3.4. The Magnitude and Copying Strategies of Child Food Insecurity in Dabat HDSS

**Figure 1:**
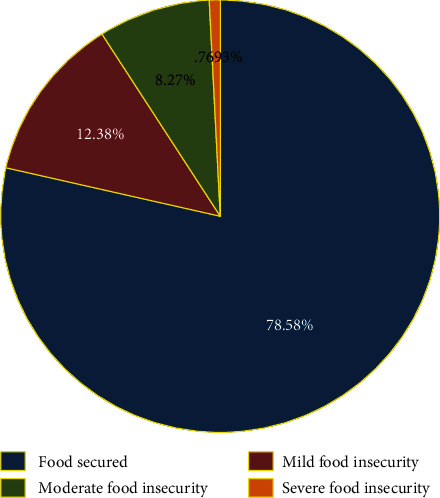
Magnitude of child food insecurity in Dabat HDSS, North West Ethiopia: April 2019.

**Figure 2:**
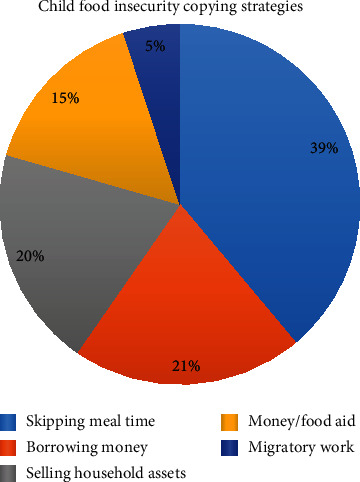
Child food insecurity copying strategies in Dabat HDSS, North West Ethiopia: April 2019.

In the study area, 1512 (21.42%) of the children were food insecure of whom about 57.8%, 38.6% and 3.6% of the children had experienced mild, moderate, and severe level of food insecurity, respectively. All most all 1391 (92%) of the mothers/caretakers of food insecure children had practiced copying strategies. More than half (57%) of them skips child meal time as child food insecurity strategy (Figures 1 and 2).

### 3.5. Factors Associated with Child Food Insecurity Level in Dabat HDSS Site

Low wealth status of the household 2.40 (2.00-2.88), heads of the household not able to read and write 2.50 (1.84-3.39), heads only able to read and write 2.56 (1.87-3.52), mothers who receive ANC care in health center 2.11 (1.56-2.58), and children who do not receive BCG vaccine were associated with the more likelihood of experiencing of different levels of child food insecurity at the univariate ordinal logistic regression model. At multivariate ordinal logistic regression, low wealth status of the household (2.26; 1.86-2.75), children who do not receive Rota III vaccine (2.26; 1.65-3.10), children with a history of initiating breastfeeding after 24 hours (2.34; 1.78-3.06), history of prelacteal feeding (2.40; 1.83-3.17), exclusive breastfeeding for more than a year (2.49; 1.045-5.93), and feeding younger children with their elders (2.40; 1.96-3.02) were factors strongly associated with the likelihood of experiencing different levels of child food insecurity (Table 4).

## 4. Discussion

In this study, 21.42% of children were food insecure and major coping strategies considered by mothers/caretakers childern under the age of five years were skipping of child meal time, borrowing money, selling of household asset, and money or food aid. All of child food insecurity coping strategies identified by this study have a potential to worsen food security; and was supported by the findings of a study among Kenyan urban poor community [21].

In our study, children from households with low wealth status were 2.26 (1.86-2.57) times and from households with medium wealth status were 1.57 (1.30-1.90) times more likely to experience mild, moderate, or severe levels of child food insecurity compared with those children who are from household with high wealth status. The more likelihood of experiencing different levels of child food insecurity by children from low and medium household wealth statuses was supported by the findings of the studies carried out in Bangladesh and Ethiopia [22–25]. This more likelihood of experiencing of different levels of child food insecurity with low and medium household wealth status might be explained by the fact households with low and medium wealth statuses might not be easily able to assure availability, accessibility, and sustainability of food for children in the household.

Illiteracy of heads of the household was strongly associated with the more likelihood of experiencing different levels of child food insecurity in this study. Children from households with heads who were unable to read and write were 1.54 (1.07-2.21) times, and those with heads only able to read and write were 1.60 (1.11-2.30) times, respectively, more likely to experience different levels of child food insecurity compared with those children from households with heads who completed secondary school education. This more likelihood of experiencing different levels of child food insecurity with the illiteracy of head of the household was supported by the findings of studies from Ethiopia conducted in households by considering children as members of the household [25–27]. This significant association between child food insecurity and illiteracy of heads of the household might be explained in the fact that illiterate heads of the household might not have better economic opportunity since being educated heads of the household is important to maintain children food security. The findings of this study had confirmed the above claim where children from households headed by those who achieve college and above educational level were 88% (47-97%) less to experience food insecurity than children from households headed by those who achieve secondary school only.

Not receiving vaccination and child illness were associated with experiencing child food insecurity. Children who does not receive BGC 2 1.77 (1.45-2.61), Rota III 2.26 (1.65-3.10), PCVII 2.22 (1.24-3.96), and PCVIII 1.82 (1.22-2.73) were found to be more likely to experience mild, moderate, or severe levels of child food insecurity than their counterparts. This significant association between child food insecurity and none receiving of child vaccine could probably be related with the more likelihood of experiencing child health problem that could have negative effect on child food security as evidenced by studies in South Africa and south-west and south Ethiopia [28–30].

Similarly, child illness and home treatment were factors associated with different levels of child food insecurity. Children with illness in two weeks of the survey were 1.32 (1.16-1.49) and those received home treatment for the illness were 2.74 (1.75-4.28) times more likely to experience mild, moderate, or severe child food insecurity in this study compared with children who had no illness with two weeks of the survey. This strong association between childhood illness and home treatment with child food insecurity could probably be explained by the fact that those children with health problems and who get treated in home might have good outcome of the phenomenon that negatively affects food security as evidenced by household food insecurity studies in America, Canada, Malaysia, and Ethiopia [18, 31–33].

Furthermore, feeding practice, feeding style, and birth order of the children were also associated with experiencing different levels of child food insecurity in this study. Children with a history of late breast feeding initiation were 1.47 (1.17-1.85) times, those who do not receive colostrum were 2.40 (1.82-3.17) times, those with a history of prelacteal feeding were 2.40 (1.82-3.17) times, those who have had exclusive breast feeding for more than six months were 1.90 (1.04-3.50) times, and those who were introduced with the supplementary food before six months were 2.36 (1.10-5.07) times more likely to experience different levels of child food insecurity than their counterparts. It was supported by the evidences of studies conducted in America and Ethiopia [18, 31]. On the other hand, children who had three meals per day were 33% (14-47%) less likely to experience child food insecurity in this study compared with their counterparts.

When we look at child feeding style, children who were fed with adults and older children were 1.64 (1.32-2.06) and 2.40 (1.90-3.02) times more likely to experience different levels of child food insecurity than their counterparts. This more likelihood of child food insecurity with feeding style may probably be due to the inability of younger children to compete with adults and older children and resulted in child food insecurity.

Children at the fourth birth order and fifth birth order were 1.40 (1.12-1.75) times and 1.28 (1.05-1.55) times more likely to experience mild, moderate, and severe levels of child food insecurity compared with children with the first birth order. This may be explained by the fact that children at the highest birth order would probably not receive care directly from their mothers as evidenced by the findings of this study where children who receive care from grandparents and other family members were 2.35 (1.53-3.59) times more likely to experience food insecurity. If grandparents or other family members provide child care due to maternal illness, it has direct effect on the worsening of child food insecurity as evidenced by the findings of different studies [19, 34].

## 5. Conclusion and Recommendation

A large segment of under-five children were experiencing mild, moderate, and severe levels of food insecurity associated with poor wealth of the household, illiteracy of the head of household, child immunization, and child feeding practices were positively associated with child food insecurity. The parents of children had practiced food insecurity copying strategies that could be probable risks for child malnutrition. Therefore, improvement of household wealth, education of the head of the household, and expansion of maternal and child health services would improve child food security.

## Figures and Tables

**Table 1:** Sociodemographic characteristics of study participants in Dabat HDSS site, Dabat district, North West Ethiopia: April 2019.

Variables	Category	Frequency	Percentage
Educational status of head of the household	Not able to read and write	3,777	53.49
	Read and write	1,811	25.64
	Primary school	696	9.85
	Secondary school	462	6.54
	College and above	155	2.20
	Not specified	160	2.26

Marital status of head of the household	Underage	259	3.67
	Married	5,419	76.75
	Single	405	5.74
	Divorced	812	11.50
	Others	166	2.35

Ethnicity of head of the household	Amhara	6,972	98.74
	Tigray	21	0.30
	Others	68	0.96

Religion of head of the household	Orthodox Christian	6,786	96.11
	Muslim	211	2.99
	Others	64	0.91

Occupation of head of the household	Under age	457	6.47
	Farmer	3,362	47.61
	Merchant	1,967	27.86
	Government employee	283	4.01
	Private employee	484	6.85
	Housewife	201	2.85
	Student	63	0.89
	Others	244	3.46

Wealth status of the household	Low	2402	34.02
	Medium	3116	44.13
	High	1543	21.85

Household family size	One	264	3.75
	Two	711	10.07
	Three	1353	19.17
	Four	1264	17.91
	Five	1180	16.72
	Six and above	2286	32.38

Residence of the child	Rural	5684	80.50
	Urban	1377	19.50

Sex of the child	Male	3,494	49.48
	Female	3,567	50.52

Age of the child	6-12 months	1,055	14.94
	13-2 4 months	1,551	21.97
	25-36 months	1,562	22.12
	37-48 months	1,669	23.64
	49-50 month	1,224	17.33

Birth order of the child	First	1,354	19.18
	Second	1,168	16.54
	Third	1,080	15.29
	Forth	1,046	14.81
	Fifth and above	2,413	34.18

Birth interval of the child	One year	241	3.41
	Two years	1,463	20.72
	Three years	2,667	37.77
	Four years	1,258	17.82
	Five and above years	1,432	20.28

Caretaker of the child	Mother	6,747	95.55
	Grandparent	222	3.15
	Others	92	1.30

Source of food item	Garden	5242	74.24
	Market	1819	25.76

**Table 2:** Maternal and child health service utilization and child feeding practice in Dabat HDSS site, Dabat district, North West Ethiopia: April 2019.

Variables	Category	Frequency	Percentage
ANC visit during last pregnancy	Yes	4650	65.86
	No	2,411	34.14

No. of ANC visit	One visit	251	5.4
	Two visits	870	18.7
	Three visits	1,682	36.18
	Four visits	1,374	29.56
	Five and above	472	10.16

Place of ANC visit during last pregnancy	Hospital	472	10.57
	Health center	4,075	87.63
	Others	83	1.80

TT vaccine during last pregnancy	Yes	3,578	76.95
	No	953	20.50
	Do not remember	119	2.54

Iron supplementation during last pregnancy	Yes	4,115	88.49
	No	535	11.51

Vit A supp. during last pregnancy	Yes	373	8.02
	No	4277	91.98

Place of delivery during the last pregnancy	Home	4,929	69.81
	Health post	66	0.94
	Health center	1,705	24.15
	Hospital	269	3.80
	Others	92	1.30

PNC service during last pregnancy	Yes	0	0
	No	7,061	100.00

Child vaccinated	Yes	6,755	95.67
	No	306	4.33

BCG vaccine	Yes	5,287	78.27
	No	1,468	21.73

Polio 0	Yes	2,089	30.92
	No	4,666	69.08

Polio 1	Yes	6,452	95.52
	No	303	4.48

Polio 2	Yes	6,229	92.01
	No	526	7.99

Polio 3	Yes	5,487	81.23
	No	1,268	18.77

Penta1	Yes	6,229	92.21
	No	526	7.79

Penta2	Yes	6,031	89.28
	No	724	10.72

Penta3	Yes	5,155	76.31
	No	1,600	23.69

Rota 1	Yes	2,621	38.80
	No	4,134	61.20

Rota 2	Yes	2,374	35.15
	No	4,381	64.85

Rota 3	Yes	1,178	17.44
	No	5,577	82.56

PCV1	Yes	3,746	55.45
	No	3,009	44.55

PCV2	Yes	3,615	53.51
	No	3,140	46.49

PCV3	Yes	3,206	47.46
	No	3,549	52.54

Measles	Yes	4,318	63.92
	No	2,437	36.08

Vit A in the last 12 months	Yes	3,649	54.01
	No	2,982	44.15
	Not remembered	124	1.84

Deworming in the last 6 months	Yes	2,616	37.05
	No	4,356	61.69
	Not remembered	89	1.26

Ever breast feeding	Yes	6,957	98.53
	No	86	1.22
	Not remembered	17	0.25

History of initiation of BF	Within one hr	4,136	58.58
	1-24 hrs	1,806	25.58
	After 24 hrs	1,045	14.79
	Do not remember	74	1.05

Fate of colostrum	Given for baby	4,828	68.37
	Discoursed	2,093	29.64
	Not remembered	140	1.99

History of prelacteal feeding	No	5,796	82.08
	Yes	1,189	16.84
	Do no remembered	76	1.08

Period of exclusive BF	<6 months	541	7.67
	6 months	4,191	59.35
	7-12 months	2,141	30.32
	>1 year	188	2.66

Currently on BF	Yes	3,783	53.57
	No	3,278	46.43

Period of BF	≤1 year	670	9.49
	13-23 months	834	11.81
	Two years	2,231	31.60
	>2 years	3,326	47.10

Age at introduction of supplementary food	Before 6 months	670	9.49
	At 6 months	4,035	57.14
	7-11 months	991	14.04
	At one year	1,135	16.07
	After one year	230	3.26

Frequency of eating per 24 hrs of the last day	1-2 times	959	13.58
	3 times	2,243	31.77
	4 times	1,979	28.03
	>5 times	1,879	26.61

Ways of child feeding	Alone	1,662	23.54
	With family	5,399	76.46

Ways of child food preparation	With adult's food	1,1719	24.34
	With old children's food	990	14.02
	Alone	4,305	60.97
	After adult's food	38	0.54
	Before old children's food	9	0.13

**Table 3:** Child hood health problems in Dabat HDSS site, Dabat district, North West Ethiopia: August 2019.

Variable	Response	Frequency	Percentage
Illness within the last two weeks	Yes	2,171	30.75
	No	4,890	69.25

Respiratory tract infection	Yes	577	26.31
	No	1594	73.69

Bloody diarrhea	Yes	167	7.6
	No	2004	92.4

Watery diarrhea	Yes	673	30.69
	No	1,498	69.31

Febrile illness	Yes	1,128	51.44
	No	1043	48.56

Ear discharge	Yes	54	2.46
	No	2117	97.54

Skin infection	Yes	103	4.70
	No	2068	95.30

Treatment for illness	Yes	517	23.57
	No	1664	76.43

Place of treatment	Home treatment	196	37.91
	Health center	301	58.22
	Traditional healers	16	3.09
	Others	4	0.77

**Table 4:** Crude and adjusted ordinal logistic regression table of child food insecurity level in Dabat HDSS site, North West Ethiopia: April 2019.

Level of child food insecurity			
Variable	Category	COR CI 95%	AOR CI 95%

Wealth states of the household	Low	2.40 (2.00-2.88)^∗^	2.26 (1.86-2.75)^∗∗^
	Medium	1.62 (1.35-1.95)^∗^	1.57 (1.30-1.90)^∗∗^
	High	1.00	1.00

Educational status of head of the household	Not read and write	2.50 (1.84-3.39)^∗^	1.54 (1.07-2.21)^∗∗^
	Read and write	2.56 (1.87-3.52)^∗^	1.60 (1.11-2.30)^∗∗^
	Primary school	1.88 (1.32-2.68)^∗^	1.57 (1.06-2.32)^∗∗^
	Secondary school	1.00	1.00
	College and above	0.11 (0.026-0.46)^∗^	0.12 (0.03-0.51)^∗∗^

Occupation of head of the household	Under 10 years old	1.50 (1.02-2.22)^∗^	1.03 (0.67-1.58)
	Farmer	1.28 (0.92-1.78)^∗^	0.96 (0.66-1.39)
	Merchant	1.66 (1.18-2.33)^∗^	1.18 (0.82-1.72)
	Private employee	0.55 (0.35-0.85)^∗^	0.59 (0.36-0.97)^∗∗^
	Gov.t employee	1.00	1.00
	Housewife	1.07 (0.66-1.74)	0.87 (0.52-1.47)
	Student	0.99 (0.47-2.10)	0.86 (0.38-1.92)
	Others	0.58 (0.35-0.97)^∗^	0.49 (0.23-1.07)

Age of the child	6-12 months	1.00	1.00
	13-24 months	1.07 (0.87-1.31)	1.14 (0.90-1.42)
	25-36 months	1.10 (0.90-1.35)	1.15(0.92-1.44)
	37-48months	1.17 (0.96-1.43)	1.16 (0.93-1.45)
	49-50 month	1.54 (1.25-1.89)^∗^	1.57 (1.25-1.97)^∗∗^

Birth order of current child	First order	1.00	1.00
	Second order	1.16 (0.94-1.42)	1.11 (0.88-1.38)
	Third order	1.20 (0.98-1.48)	1.04 (0.82-1.30)
	Fourth order	1.55 (1.27-1.90)^∗^	1.40 (1.12-1.75)^∗∗^
	Fifth and above	1.36 (1.15-1.62)^∗^	1.28 (1.05-1.55)^∗∗^

Place of ANC visit during last pregnancy	Hospital	1.00	1.00
	Health center	2.11 (1.56-2.85)^∗^	1.86 (1.33-2.59)^∗∗^
	Others	1.87 (1.01-3.46)^∗^	1.06 (0.52-2.18)

TT vaccine during last pregnancy	Yes	1.00	1.00
	No	1.49 (1.25-1.76)^∗^	1.26 (1.04-1.53)^∗∗^
	No remembered	1.16 (0.73-1.82)	1.55 (0.94-2.57)

Place of delivery	Home	1.60 (1.14-2.25)^∗^	1.53 (1.01-2.33)^∗∗^
	Health post	0.69 (0.29-1.62)	0.58 (0.19-1.77)
	Health center	1.29 (0.90-1.84)	1.25 (0.83-1.90)
	Hospital	1.00	1.00
	Others	1.20 (0.63-2.29)	1.35 (0.61-2.94)

BCG vaccine	Yes	1.00	1.00
	No	2.95 (2.58-3.36)^∗^	1.77 (1.45-2.16)^∗∗^

Polio 0	Yes	1.00	1.00
	No	1.19 (1.04-1.36)^∗^	0.90 (0.73-1.11)

Penta3	Yes	1.00	1.00
	No	0.83 (0.71-0.96)^∗^	0.52 (0.40-0.67)

Rota 1	Yes	1.00	1.00
	No	1.63 (1.43-1.86)^∗^	1.16 (0.75-1.79)

Rota 2	Yes	1.00	1.00
	No	1.67 (1.46-1.91)^∗^	0.67 (0.42-1.07)

Rota 3	Yes	1.00	1.00
	No	2.58 (2.11-3.16)^∗^	2.26 (1.65-3.10)^∗∗^

Measles	Yes	1.00	1.00
	No	0.79 (0.70-0.90)^∗^	1.07 (0.87-1.31)

PCV1	Yes	1.00	1.00
	No	1.84 (1.63-2.08)^∗^	0.60 (0.35-1.06)

PCV2	Yes	1.00	1.00
	No	1.95 (1.73-2.21)^∗^	2.22 (1.24-3.96)^∗∗^

PCV3	Yes	1.00	1.00
	No	2.04 (1.80-2.32)^∗^	1.82 (1.22-2.73)^∗∗^

Vitamin A supp. in the last 12 months	Yes	1.00	1.00
	No	0.45 (0.40-0.52)^∗^	0.30 (0.24-0.37)^∗∗^
	Not remembered	1.51 (1.05-2.18)^∗^	1.14 (0.67-1.94)

Deworming in the last 6 months	Yes	1.00	1.00
	No	1.29(1.14-1.47)^∗^	1.43 (1.19-1.73)^∗∗^
	Not remembered	1.38 (0.84-2.28)	1.26 (0.62-2.58)

History of initiation of breast feeding	Within one hr	1.00	1.00
	1-24 hrs	1.34 (1.17-1.54)^∗^	1.47 (1.17-1.85)^∗∗^
	After 24 hrs	1.94 (1.66-2.27)^∗^	2.34 (1.78-3.06)^∗∗^
	Do not know	0.73 (0.37-1.43)	0.29 (0.05-1.44)

Fate of colostrums	Given for baby	1.00	1.00
	Discarded	1.41 (1.25-1.60)^∗^	1.32 (1.07-1.64)^∗∗^
	Not remembered	0.71 (0.44-1.15)	0.98 (0.42-2.25)

History of prelacteal feeding	Yes	0.61 (0.50-0.72)	2.40 (1.82-3.17)^∗∗^
	No	1.00	1.000
	Do not know	0.49 (0.24-0.98)^∗^	1.14 (0.29-4.47)

Period of exclusive breast feeding	<6 months	0.81 (0.63-1.03)^∗^	0.42 (0.20-0.86)
	6 months	1.00	1.00
	7-12months	1.18 (1.04-1.34)^∗^	1.90 (1.04-3.50)^∗∗^
	>1 year	1.82 (1.30-2.55)^∗^	2.49 (1.04-5.93)^∗∗^

Period of breast feeding	≤1 year	1.11 (0.78-1.56)	0.99 (.67-1.46)
	13-23 months	1.13 (0.82-1.54)	1.11 (0.80-1.55)
	2 years	1.00	1.00
	>2 years	1.61 (1.32-1.98)^∗^	1.49 (1.20-1.86)^∗∗^

Age at introduction of supplementary food	≤6months	1.09 (0.88-1.37)	2.36 (1.10-5.07)^∗∗^
	At 6months	1.00	1.00
	7-11months	1.01 (0.85-1.21)	0.60 (0.33-1.10)
	At one year	1.17 (1.00-1.37)^∗^	0.56 (0.29-1.06)
	After one year	1.75 (1.31-2.34)	0.73 (0.31-1.72)

Frequency of feeding	1-2 times	0. 58 (0.47-0.72)^∗^	0.99 (0.56-1.75)
	3times	0.61 (0.52-0.71)^∗^	0.67 (0.53-0.86)^∗∗^
	4 times	1.00	1.00
	≥5times	0.81 (0.70-0.95)^∗^	0.85 (0.68-1.06)^∗∗^

Ways of preparing child food	Alone	1.00	1.00
	With family	1.82 (1.56-2.12)^∗^	1.40(0.68-2.87)

Ways of child feeding	With adults	1.54 (1.34-1.77)^∗^	1.64 (1.32-2.06)^∗∗^
	With old children	2.13 (1.82-2.49)^∗^	2.40 (1.90-3.02)^∗∗^
	Alone	1.00	1.00
	After adults	1.34 (0.58-3.09)	3.19 (1.18-8.66)^∗∗^
	Before old children	0.85 (0.10-7.20)	6.33 (0.35-112.69)

Caretaker of the child	Mother	1.000	1.00
	Grandparent	1.46 (1.08-1.97)^∗^	2.35 (1.53-3.59)^∗∗^
	Others	1.53 (0.95-2.45)	1.93 (1.02-3.67)^∗∗^

Child illness in two weeks of survey	No	1.00	1.00
	Yes	1.32 (1.16-1.49)^∗^	1.42 (0.23-1.43)

Watery diarrhea	Yes	0.63 (0.49-0.79)^∗^	1.11 (0.69-1.78)
	No	1.00	1.00

Treatment for illness	Yes	1.00	1.00
	No	0.58 (0.45-0.74)^∗^	0.36 (0.12-1.12)

Place of treatment	Home	2.19 (1.47-3.27)^∗^	2.74 (1.75-4.28)^∗∗^
	Health institution	1.00	1.00
	Traditional healer	1.81 (0.65-5.02)	2.55 (0.71-9.19)

^∗∗^Factors associated with child food insecurity at *p* value < 0.005.

## Data Availability

The data used to support the findings of this study are available from the corresponding author upon request.

## Additional Points

*Limitation of the Study*. Data was collected only from the mothers/caretakers, where involvement of both parents may better supplement the evidence. *Strength of the Study*. For the assessment of mothers/caretakers' food preference, the study considers the relatively large sample size and advanced statistical model.
